# Effective risk management in the shadow of COVID-19 pandemic: The evidence of Indonesian listed corporations

**DOI:** 10.1016/j.heliyon.2023.e15744

**Published:** 2023-04-29

**Authors:** Jun Huang, Bienmali Kombate, Yun Li, Konan Richard Kouadio, Peijun Xie

**Affiliations:** School of Business Administration, Hunan University, Changsha City, Hunan Province, 410082, PR China

**Keywords:** Risk management, Non-financial corporation, Operational efficiency, COVID-19 pandemic, Solvency, And liquidity

## Abstract

The study uses COVID-19 to identify the treatment group as the difference in change of non-financial corporations (NFCs) risk management ratios over time to investigate the causal effect of the NFCs' effective risk management (ERM) practices on operational efficiency (OE). ERM was measured by solvency and liquidity ratios, while the risk management theory was developed to refine the scope of the study. The data were collected from the central bank of Indonesia to map the empirical analysis, and the difference in difference (DID) technique was used to illustrate how NFCs react to mitigate the negative impact of COVID-19 and generate OE. Specifically, a quasi-natural experiment was used to size the effect of ERM practices on corporate OE during the COVID-19 pandemic. The descriptive analysis revealed that the COVID-19 pandemic effect has been unequal across different industrial sectors. Moreover, the empirical findings showed that corporate risk management during COVID-19 is the source of structural change, which affects its existence and operational efficiency. While debt amount and age may affect corporate credit score, ERM practices led the indebted corporation to the flexibility of debt refinancing or/and restructuring, which offers them the ability to prevent bankruptcy and adapt to the changes while operating efficiently. The finding revealed evidence of the important role of long-term debt in offering protection to NFCs during the credit supply shock brought in by the COVID-19 pandemic. Furthermore, the findings show that long-term debt is negatively associated with corporate OE. This was expected given that corporations use long-term debt financing for long-term investment, while short-term debt funds the working capital. Thus, to assess the effect of debts on corporate OE, managers should consider their maturity structure, among other factors.

## 1 Introduction

Public concern has grown substantially about intensified corporate financial risk arising from the credit supply shock driven by the COVID-19 pandemic's impact on the financial system. Financial risk triggers the inefficiency of corporate operations, traumatizes business activities, and inflicts unprecedented harm to the economy. Previous studies have shown that corporations use debt financing to improve operational efficiency [[1], [2], [3], [4]]. In addition, there is a limitation to using internal resources as sources of financing because inflation erodes the equity base of the business over time, and management may not be able to provide additional funds to meet the increased working capital requirement even where growth is curtailed [5,6].

However, during the COVID-19 pandemic, accessing capital through debt financing has become difficult. At the same time, many governments, in collaboration with their central banks, responded proactively to this crisis with a package of macroeconomic and financial measures to attempt to support the banking industry's resilience. For example, the moral hazard issues notwithstanding [7], policy instrument was used as an alternative policy because of its effectiveness in terms cost by many governments in response to mitigate the negative impact of COVID-19 [8]. Particularly, debt servicing, amortization relief, debt restructuring, and debt refinancing measures have been put into place by governments in some most affected countries and are considered similar to demand and supply stimulus measures by increasing disposable income for NFCs [9]. Yet, the lockdown measures and consequences have created increased concern for the financial system stability leading to the credit shock supply [10]. In addition, empirical evidence also suggested that government macroeconomic and financial measures for economic recovery during a crisis are not associated with improving corporate liquidity capability [11].

In the setting of the COVID-19 pandemic, it is important to note that a plethora of empirical studies acknowledged the negative impact of this pandemic on business activities. However, most of them narrowed their investigation to conclude that the pandemic has deteriorated NFCs' balance sheets and therefore increased their financial risk and bankruptcy [[12], [13], [14], [15]]. In the meantime, even the government measures do not generate the immediate generalized effect that recovery policies should entail, and given everything, NFCs stood in the business thanks to sophisticated risk management practices. Nevertheless, no empirical study has attempted to investigate the causal effect of these ERM practices on continual corporate existence during the COVID-19 pandemic. Hence, this study addressed the research gaps in the existing literature by identifying the treatment group as the difference in changes over time (pre-treatment and post-treatment) across the two groups (treatment and control).

In order to clarify this further, despite the fact that COVID-19 has deteriorated NFCs' balance sheets and increased their financial risk and bankruptcy [16], this paper does not oppose that debt amount and maturity may affect corporate credit score [17]. But rather, it examines how indebted NFCs, through their ERM practices, benefit from the temporary regulatory relief policies implemented by the government to help restart economic systems after the deep crisis brought on by the lockdown. The paper, therefore, opens a debate on the role of debt servicing, amortization relief, debt restructuring, and debt refinancing measures during the credit shock supply driven by COVID-19, which has also been driven by the problems encountered in many economies by commercial and development banks in terms of non-performing loans and the selection criteria used in allocating funds. This demonstrates that this paper could be a reference material for practitioners, and governments, in cooperation with their central and commercial banks, to speed up the economic recovery just in case future crises of this type happen worldwide.

In addition, this study is a complement to the existing literature on the effect of the COVID-19 pandemic on NFCs, which suggested that the pre-COVID-19 corporate characteristic, including cash (solvability and liquidity), profitability, and corporate governance, shape corporate resilience and performance with the COVID-19 pandemic [[18], [19], [20], [21]]. The study focuses instead on the causal effect of corporate Effective Risk Management practices during the post-treatment on its Operational Efficiency. Relying on the macro-level panel data, we analyze how NFCs react to mitigate the negative impact of COVID-19 and continuity in business. The paper will not have anything to say about the corporate’ characteristics since the dataset doesn't provide micro-level information, nor the most recent situation in the latter part of March 2021, since the data do not cover this period.

The study measured corporate ERM by its solvency and liquidity. However, relying on [22], who argued that efficiency means “doing the right things,” the study assumed that ERM during the period of post-treatment is associated with corporation OE. To verify this assumption, the study applied a difference in difference (DID) technique to demonstrate how NFC reacts to mitigate the negative impact of COVID-19 and generate OE, especially, a quasi-natural experiment was used to size the ERM effect on OE during the COVID-19 pandemic. In particular, the study assesses how the solvability and liquidity of Indonesian-listed corporations from different industrial sectors change affect their OE following the COVID-19 pandemic period.

**Fig. 1:**
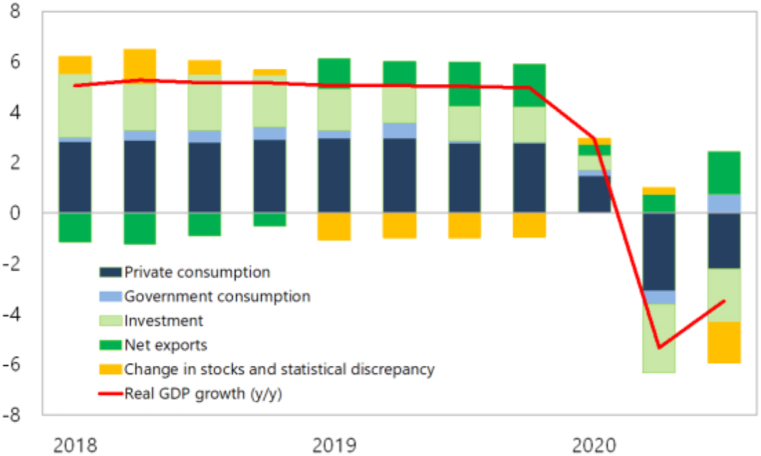
Contribution to the real GDP growth (in % YoY). Source: CEIC Data Co. Ltd., and IMF data estimates.

Indonesia is one of the economies in the world that faces a hard outlook during the COVID-19 pandemic, as its economy has been shocked by a major episode of capital outflows [23]. The key economic risk is not the old one of a reverse in capital flows prompting a currency crisis in the Asian Financial Crisis of the late 1990s. About 1.8 million people became unemployed between February 2020 and 2021, and 2.8 million individuals fell into poverty [[24], [25], [26]]. The fundamental problem is an internal issue financing a budget deficit large enough to afford adequate health expenses and financial support to caution what is likely the most severe global economic downturn since the great depression. Additionally, its dependence on foreign capital inflows has so far been a weak point as it was one of the worst impacted by the mass exodus of foreign capital from emerging markets since the COVID-19 health crisis became a global pandemic, and more than US$10 billion was withdrawn from its capital markets; besides the rupiah plunged at one point by almost 20% [27]. The succession of these events led the country, after many years of robust growth, the GDP drop to 2.1% in 2020 (see Fig. 1) [9].

While large, this recession was smaller than in other economies in the Asia-Pacific region, reflecting a small number of stringent containment measures and lower dependence on highly impacted sectors. The recent World Bank report shows that the country's credit growth has fallen (from 7.95 in April 2020 to −2.4% in December 2020), leading to a moderate corporate vulnerability due to a mixture of credit demand and supply constraints, such as weak effectiveness of monetary policy transmission, low take-up of risk-sharing mechanisms such as guarantees, as well as historically low reliance on banking credit among micro, small and medium enterprises (MSMEs) [28]. Yet, the pandemic risks still loom large, with cases increasing significantly; the country remains vulnerable to new waves determined by more contagious strains as experienced by other countries and potentially higher mobility and viral spread during festivities [24]. Moreover, Habir and Wardana [29] revealed that the non-performing loans (NPLs) continue to creep up, and a prolonged pandemic would adversely affect economic recovery and further erode the banking sector's capital levels. Given everything, the country's non-financial corporate stood in the business thanks to sophisticated risk management practices.

The remainder of the paper is structured as follows. Section 2 gives an overview of the relevant theory and literature. Section 3 describes the study variables, data and research strategy, and the research model estimation. Section 4 reports the study's empirical results, and section 5 concludes with study limitations.

## 2 Literature review

Many theories investigated liquidity and solvency (corporate financial risk indicators) management, among others. The shiftability theory proposed by Ref. [30] emphasized that if commercial banks maintain a substantial amount of assets that can be shifted to the other banks for cash without a material loss in case of need, then there is no necessity to rely on maturities. i.e., an asset to be perfectly shiftable must be instantly transferable without capital loss when the necessity for liquidity rises. This theory was not applicable to the current study due to the specific context of the COVID-19 pandemic's negative economic impact [31].

Another theory that debates liquidity and solvency management is the liability management theory developed in 1960. This theory suggests there is no need to follow old liquidity norms like keeping liquid assets and liquid investments, among others [32]. According to this theory, a corporate can satisfy its liquidity needs by borrowing money in capital markets. However, this theory could not be applicable due to the negative impact of the COVID-19 pandemic on the financial market [33,34].

Therefore, as pointed out by Ref. [35], there are clear elements that can explain the Importance of the use of risk management theory in the context of the present study. First of all, the increasing solvency risk facing the Indonesian corporation due to the COVID-19 pandemic may force them to adopt at least some level of risk awareness. On the other hand, with the government's new policies for economic recovery due to COVID-19, corporations are generally faced with legal requirements for the application of some integral sophisticated risk management practices [9]. The elements described generate new risks and increase the impact of existing risks [36,37]. Thus, the modern recognition of risk management as a procedure that is complementary and integrated with other corporation processes, continuously and formally, can be a relevant approach to the reality facing the organization. In this sense, the risk management process is not only an instrument to prevent and manage the impact of damaging events on the corporation but also a power to see opportunities [35].

Effective risk management against liquidity and solvency risks is crucial and cannot be refuted as far as the sustainability of any corporation in the long term is concerned. It includes identifying, assessing, and prioritizing risks about any institution, entity, or corporation aspect. This would be followed by a harmonized and economic employment of resources to lessen, control, and monitor the impact and possibility of the unfortunate occurrence [38]. It is an ongoing process that requires the corporation to apply adequate security measures to diminish the threats. It helps NFCs match the maturity of their assets and liabilities and offers protection from credit supply shocks, for example, having to restructure and/or refinance long-term debt in bad situations.

Risk evaluation should be a compulsory step, as well as determining their probability of doing so. It refers to the process of probing what might cause damage during the period of activity to see whether sufficient safety measures have been taken to minimize the risk as much as possible or whether supplementary control measures are needed to prevent danger [39]. Thus, the present study's purpose of investigating the causal effect of ERM on the operational efficiency of the Indonesian-listed NFCs during the COVID-19 pandemic and applies this theory that helps to discuss liquidity and solvency risks, thus, being part of another resource that will increase the awareness of managers and stakeholders with corporation ERM.

It has been widely documented that a corporation's ERM practices are an important management tool that drives its OE [[40], [41], [42], [43], [44], [45], [46]]. While debts are used to finance corporate operations and are important for its capital structure as managers may decide the usage of debts, that is, whether and to what degree they will finance short or long-run inputs, the availability of and access to credit significantly impacts the OE of businesses [34,[47], [48], [49], [50]]. Champion [51] added that leverage is the way to improve corporate OE. Debt financing plays a significant role in improving corporate productivity and is crucial for its future growth [52]. Debt is a critical source of funds for most corporations accounting for over 90% of all new external financing [53,54].

In addition [55], pointed out that corporate use credit issuance to improve their OE and lower the costs related to the separation of control and ownership. This finding was later supported by scholars (such as [56,57]) who concluded that debt issuance is an appropriate instrument to improve corporate OE and manager risk management action. Moreover, several empirical studies have documented that OE is driven by corporate credit issuance [[58], [59], [60], [61], [62]]. Lyu and Chen [63] also added that debt financing has a disciplinary effect if ownership and management are separated, as is usually the case in large corporations. Debt financing increases the pressure on managers, encouraging them to perform more efficiently.

A growing number of empirical studies emphasized that the impact of debt on corporate OE relies on their maturity (short-term or long-term debt). Some scholars [64,65] argued that corporate shortens their credit maturity to execute credit repayment on the business for several reasons. Among them are reducing the interest rates on loans, changing the loan structure, consolidating debts, and freeing up cash. This measure matches the conclusion of [66] that debt refinancing and/or restructuring is associated with corporate OE in the use of the asset. Moreover, several empirical studies show that short-term debt has a significant and positive effect on corporate OE [56,57,[67], [68], [69], [70], [71]]. However, from the corporate perspective, long-term debt offers protection from credit supply shocks and from having to restructure and/or refinance in bad times, facilitating long-term investments and improving corporate OE, which also buffers corporate managers from the regular monitoring that short-term debt requires as it comes up for renewal [42,46,72,73].

The world bank global financial development report suggests that the use of long-term debt improves corporate OE with developed financial institutions and markets and the capability to enter into long-term contracts; corporations can grow at faster rates than they could achieve by depending on short-term credit alone [74]. Consistent with these results [75,76], suggest that the differences in corporate debt maturity had real important effects during the financial crisis of 2008. Although government subsidies and directed credit can lengthen the maturity structure, there is no evidence that such steps are associated with better corporate OE [11,74,[77], [78], [79], [80], [81]]. It is important to note that short-term debts are used for working capital, and long-term debt is often used to make long-term investments, such as purchases of fixed assets or equipment [82,83].

Another issue is that the maturity of the long-term debt corresponds to the maturity of cash flows from corporate investment and which must be refinanced on terms that depend on the credit rating at the time of its renewal, whereas short-term debt matures before the cash flows reach from a corporate investment [84]. This clearly emphasizes that the analysis of the implication of debt (short-term and long-term) on corporate OE should consider the maturity of the investment they entail. This was further supported by the empirical study of [42], who argued that long-term debt and the possible adverse consequences when it is in short supply are somewhat at odds with recent theoretical contributions that highlight the fact that the use of short-term debt may be associated with higher-quality NFCs and may have better incentive properties. As short-term debt (liquidity) is preferable for working capital [85], OE will be negatively associated with long-term debt financing [84]. In other words, all things being equal, NFC that has enough liquidity to fund its operation (working capital) will not use long-term debt to fund its working capital [86]. Table 1 Provides a summary of key studies.

**Table 1:** Summary of some key studies.

Author(s)	Main Objective(s)	Argument(s) or Conclusion(s)
Moulton [121]	The problem of liquidity.	There is no need to rely on maturities since commercial banks'' assets can be transferred to the other banks for cash without a material loss in case of need for liquidity.
Roussakis [122]	Management of commercial banks through the formulation and implementation of sound and flexible policies	There is no need to follow old liquidity norms like keeping liquid assets and liquid investments since commercial bank can satisfy their liquidity needs by borrowing money in capital markets.
Padovani[123]	Management of financial risk.	Risk management is not only an instrument to prevent and manage the impact of damaging events on the corporation but also a power to see opportunities.
Kombate [38]	Importance of management control system.	Risk management is a harmonized and economic employment of resources and helps to lessen, control, and monitor the impact and possibility of an unfortunate occurrence.
Kemshall [39]	Risk rationalities.	Risk management is a process of probing what might cause damage during the activity period and determining whether sufficient safety measures have been taken to minimize it as much as possible or whether supplementary control measures are needed to prevent it.
Nazir et al. [46]	Importance of debts on corporate OE.	The availability of and access to credit significantly impacts corporate OE.
Champion [51]	Impact of leverage on corporate OE.	Leverage is the way to improve corporate OE.
Aziz et al. [52]	Importance of debt financing on corporate growth.	Debt financing plays a significant role in improving corporate productivity and is crucial for its future growth.
Jensen [66]	Relationship between debt and OE.	Corporations use credit issuance to improve their OE and lower the costs related to the separation of control and ownership.
		Debt refinancing and/or restructuring is associated with corporate OE.
Barclay et al. [64]	Relationship between debt maturity and OE.	Corporations shorten their credit maturity to execute credit repayment on the business for several reasons: reducing the interest rates on loans, changing the loan structure, consolidating debts, and freeing up cash.
Alcock et al. [71]	Relationship between short-term debt and OE.	Short-term debt has a significant and positive effect on corporate OE.
Jaramillo et al. [42]	Importance of long-term debt.	Long-term debt offers protection from credit supply shocks and from having to restructure and/or refinance in bad times, facilitating long-term investments and improving corporate OE.
Bruhn [74]	Impact of long-term debt on corporate growth.	Using long-term debt can help corporates grow faster than they could achieve by depending on the short-term debt alone.
Lalinsky et al. [81]	Government subsidies and directed credit effect on corporate OE.	Government subsidies and directed credit have a limited effect on improving corporate OE.
Demirgüç-Kunt et al. [82]	Difference between short-term and long-term debt.	Short-term debts are used for working capital, and long-term debt is often used to make long-term investments, such as purchases of fixed assets or equipment.
Legesse et al. [84]	Analyzing the effect of debt maturity and corporate OE.	The maturity of the long-term debt corresponds to the maturity of cash flows from corporate investment, but short-term debt has a maturity before the cash flows reach corporate investment.
Jaramillo et al. [42]	Importance of Long-term debt.	Long-term debt and the possible adverse consequences when it is in short supply are somewhat at odds with recent theoretical contributions highlighting that the use of short-term debt may be associated with higher-quality NFCs and may have better incentive properties.
Legesse et al. [84]	Role of short-term debt in working capital.	As short-term debt (liquidity) is preferable for working capital, OE will be negatively associated with long-term debt financing.
Memon et al. [86]	Short-term VS long-term debt financing working capital effect on OE.	Corporations that have access to short-term debt to fund its operation will not use long-term debt to fund their working capital.

Generally, managerial efficiencies enhance corporate short-term debt financing ability to fund their operations and long-term debts to fund their long-term investment. Thus, the study set the hypotheses given below.

H1 Non-financial corporations' ERM practices aiming to raise short-term debt (liquidity) positively and significantly affect their OE.

H2 Non-financial corporations' ERM practices aiming to raise long-term debt (solvability) negatively and significantly affect their OE.

## 3 Materials and methodology

After screening multiple methodologies, this study has applied the Difference-in-Differences, commonly called DID method, to undertake the analysis. DID methods have become common in estimating causal relationships since the work by Ref. [87]. DID is a statistical technique that analyzes data from a nonequivalence control group design and makes a causal inference about an independent variable (e.g., policy, treatment, or an event) on an outcome variable. Yet, the present study aims to investigate the causal effect of NFCs' effective risk management on its OE during the COVID-19 pandemic; DID method is justified and appropriate in the context of this study. The basic idea is that observations are collected for two groups from two periods. One of the groups is the treatment group which is exposed to treatment in one period. The other is the control group which receives no treatment during both periods.

DID analysis technique removes biases in the second period of comparison among the treatment and control groups that might be the result of permanent differences between those groups and biases from comparison over time in the treated group that could be the result of time trends not related to the treatment [[88], [89], [90]]. In addition, DID analysis technique helps contemporary researchers to take an active approach seeking to construct comparison groups in a common trend assumption by checking the statistical tests (sensitivity analysis and robustness analysis) and graphical analysis that helps to validate the method assumptions [91].

Moreover, the DID technique is a quasi-experimental approach that compares the changes in outcomes over time between the observation that are exposed to the treatment (the treatment group) to the observations that are not (the control group) [92]. It is applied in observational situations where exchangeability cannot be assumed between the treatment and control groups [93]. Additionally, it relies on a less strict exchangeability assumption, i.e., in the absence of treatment, the unobserved differences between treatment and control groups are the same over time. Hence, it is a useful technique to apply when randomization on the individual level is not possible [94].

Moreover, DID technique offers some advantages such as (i) intuitive interpretation of the results [95], (ii) can obtain causal effect using observational data if the assumption is met [96], (iii) can use of individual or group-level data [95], (iv) the comparison groups can begin at different levels of the outcome variable (DID focuses on change rather than absolute levels), and (v) it accounts for change due to variables other than intervention [97,98]. However, it has some limitations, such as (i) it requires baseline data and a non-intervention group [99], (ii) it cannot use if intervention allocation is determined by baseline outcome [100], (iii) it cannot be applied if the groups' composition of pre and post-treatment are not stable [101], and (iv) it cannot be applied if comparison groups have different outcome trend [97,102]. Following subsections of this section details the data and technique used, the research variables, and the research model estimation.

### 3.1 Data and research strategy

The data for this study were collected from the Indonesian Central Bank “Bank of Indonesia.^1^” The dataset contains the quarterly aggregated benchmark financial ratios by industrial sector of the eight industrial sectors that comprise the Indonesian listed corporations. This included basic industry; agriculture; consumer goods; property; infrastructure, utility, and transportation; mining; miscellaneous; and trade. The sample period was from October 2018 to March 2021. This sample period provides a favorable context for research related to (1) significant changes in the financial indicator of the non-financial corporation in terms of risk and profitability segments as well as (2) regular adjustments in operation management due to the COVID-19 pandemic. The study eliminated the concern of sample selection because the dataset contains the benchmark financial indicator by industrial sector of all the industrial sectors included in Indonesian listed corporations. Table 2 presents the descriptive statistics of the study sample period.

**Table 2:** Summary Statistics of the outcome variables for the study sample Period.

	Agriculture Sector				Mining Sector				Basic Industry Sector				Miscellaneous industry Sector			
	Mean	SD	Min	Max	Mean	SD	Min	Max	Mean	SD	Min	Max	Mean	SD	Min	Max
DE	1.28	0.09	1.18	1.45	1.33	0.11	1.13	1.46	1.16	0.03	1.10	1.21	1.16	0.08	1.03	1.27
DA	0.56	0.02	0.54	0.59	0.57	0.02	0.53	0.59	0.54	0.01	0.52	0.55	0.54	0.02	0.51	0.56
CR	0.93	0.08	0.84	1.05	1.31	0.07	1.23	1.41	1.57	0.08	1.38	1.65	1.26	0.07	1.13	1.35
QR	0.63	0.07	0.54	0.72	1.12	0.08	1.03	1.26	1.09	0.05	0.98	1.14	0.95	0.07	0.83	1.06
ROA	−.95	2.09	−4.49	2.44	3.00	1.82	.96	6.38	2.48	1.17	1.4	4.90	4.13	.97	2.47	5.29
ROE	−2.29	4.93	−10.99	5.63	7.06	4.29	2.22	14.82	5.31	2.40	3.04	10.19	9.00	2.30	5.08	11.63
OE	2.37	.21	2.00	2.61	2.04	.09	1.89	2.20	2.15	.17	1.91	2.42	1.39	.17	1.17	1.67
	Consumer goods industry sector				Property Sector				Infrastructure, Utility, and transportation				Trade Sector			
	Mean	SD	Min	Max	Mean	SD	Min	Max	Mean	SD	Min	Max	Mean	SD	Min	Max
DE	0.75	0.07	0.65	0.87	1.20	0.08	1.08	1.35	1.96	0.32	1.58	2.48	0.92	0.06	0.83	1.03
DA	0.43	0.02	0.39	0.46	0.54	0.02	0.52	0.57	0.66	0.04	0.61	0.71	0.48	0.02	0.45	0.51
CR	1.90	0.10	1.70	2.09	1.58	0.11	1.39	1.70	0.66	0.06	0.58	0.74	1.41	0.05	1.35	1.51
QR	1.07	0.08	0.92	1.23	0.98	0.15	0.79	1.18	0.63	0.06	0.56	0.71	1.02	0.04	0.97	1.09
ROA	11.06	2.02	7.11	12.93	1.21	2.20	−2.38	4.03	1.68	1.57	−1.76	3.39	2.71	1.21	1.22	5.26
ROE	18.98	3.12	12.81	21.87	2.55	4.86	−5.3	8.84	4.54	4.57	−6.12	9.03	5.22	2.30	2.35	10.02
OE	2.82	.14	2.58	3.03	3.59	.50	2.97	4.32	1.39	.14	1.16	1.61	.027	.01	.012	.052

This table presents the summary statistics of the study's main variables ((benchmark financial ratios) for the study sample period (October 2018 to March 2021). All the variables are expressed in percentages (%).

On January 23, 2020, the Chinese government announced the discovery of the COVID-19 virus in Wuhan caused by severe acute respiratory syndrome coronavirus 2 (SARS-CoV-2) and decided to lockdown the city followed by the lockdown of the Hubei province and major cities and provinces in China due to the rapid spread of the virus. Indonesia, which has been criticized for the delay in response because of the rapid spread of the virus in the neighboring countries, including Malaysia and Singapore, in early February [103], announced its first case on March 2, 2020, and the WHO announced it on March 11, 2020, qualifying the COVID -19 health crisis as a pandemic. The International Air Transport Association data showed in late March 2020 that more than 156 countries had put in place some policies and measures to limit the spread of the virus, including travel restrictions, denying entry, restricting visas, or imposing mandatory quarantine, among other measures, with 104 countries closed entirely [104]. However, the Indonesian government delayed the lockdown and containment measures, arguing it has a harsh economic impact on other developing economies such as India. This led to an increase in cases and deaths, making the country the highest in Southeast Asia in early April 2020 [105]. On April 2, 2020, the government had therefore declared a national health emergency and took action by closing the international borders, followed by strict containment measures imposed on April 10, 2020, through gubernatorial Regulation 33/2020 and Decree 380/2020 to restrain the spread of the virus in Jakarta which was further implemented in other major provinces and cities [106]. However [106], added that these measures' implementation was further delayed for five weeks in some places, such as Depok, a city within the greater Jakarta metropolitan area.

These containment measures have led to an inverse and dramatic condition in the country's economy, which is discussed early in this paper. Thus, in addition to mass public testing, the government allocated Rp 87.55 trillion to the health sector within the revised Stimulus III in March for materials to empower the country laboratories to conduct polymerase chain reaction (PCR) testing [107]. For the economic recovery, the government has taken adequate macroeconomics and harmonized a policy package to the unprecedented shock measures, such as the ceiling on the country's budget deficit of 3% of GDP initially planned to be suspended through 2022. The ban on the Bank of Indonesia's acquisitions of longer-term government bonds in the prime market was detached. The specific major policies are (i) four consecutive fiscal packages aimed at consolidating healthcare and mitigating the economic and social hardship of the COVID-19 pandemic. The total provision for the COVID-19 response beneath the six subprograms of the plan for the national economic recovery (PEN) counted to 4.4% of GDP in 2020. The 2020 fiscal deficit was predicted to widen to 6.3% of GDP in the revised final budget, from 1.8% in the initial budget with an estimated fiscal urge (net of budget reallocation) of 3½% of the GDP budget. (ii) Bank of Indonesia lowered its benchmark interest rate by 125 basis points and increased liquidity support for commercial banks. It also stabilized domestic market conditions by using a “triple intervention” strategy in the spot market and domestic non-deliverables forward (DNDF) foreign exchange (FX) and sovereign bond markets in local currencies. In addition, commercial banks were allowed to spend their capital conservation buffers, while loan classification and restructuring policies have been partially relaxed. Detailed fiscal, monetary, and financial measures taken by the Indonesian government are included in Table 3.

**Table 3:** Indonesia macroeconomic measure for economy recovery.

List of Measures (Announced or Under Implementation)	Expected Size (In % GDP)	Expiration Date
**Fiscal**		
Target Support to Hard-hit Industry 1/	0.2	
Support health care to fight covid-19	0.5	
Strengthening existing social protection programs 2/	1.3	
Support for promoting restructuring and financing for MSMEs 3/	0.8	
Corporate Income tax rate reduction 4/	0.1	Permanent
Tax relief and incentives for firms and low-income households	0.6	
Capital injection and loan to State-owned enterprises	0.3	
Other support measures through local governments	0.6	
Credit guarantee on working capital loan to labor-intensive industries	0.6	
**Monetary**		
Policy rate reduction (125 bps, from 5 to 3.75%)		
“Triple intervention " to stabilize domestic financial markets Cuts in the FX and domestic reserve requirement ratios for banks		
Macroprudential liquidity buffer ratio for banks raised by 200 bps Enhanced liquidity support targeted to banks 5/		
Relaxed the macroprudential intermediation ratio		
Provision of funding to LPS for the handling of bank solvency problems		
Liquidity provision to banks and firms through term-repo transactions		
Bank of Indonesia purchases government bonds under a burden-sharing agreement		End −2020
Bank of Indonesia to act as a buyer of last resort for local-currency government bonds		End −2020
**Financial**		
Relaxed loan classification and loan restructuring procedures		Mar 2022
Measures aimed to mitigate stock market volatility 6/		
Delayed the implementation of the Basel III reform standards		Jan 2023
Postponement of mark-to-market valuation of securities for six months		Sep 2020
Allowance to use the Capital Conservation Buffer		Mar 2022
Relaxed LCR and NSFR requirements for banks 7/		Mar 2022
Relaxed rules on credit cards to support cashless transactions 8/		
Lower down payment requirements for environment-friendly vehicles		
1/For tourism and other labor-intensive industries.		
2/These include food aid, cash transfers, electricity subsidies, and unemployment benefits.		
3/These include interest subsidies, credit guarantees, and loan restructuring funds.		
4/From 25% to 22% for 2020–2021 and 20% starting in 2022.		
5/These includes introducing daily repo auctions, increasing the max duration for repo and reverse repo transaction, and increasing the frequency of FX swap auctions.		
6/These included the prohibition of short selling and allowing the listed corporation to buy back without a prior shareholders' meeting		
7/LCR and NSFR stand for Liquidity Coverage Ratio and Net Stable Funding Ratio, respectively.		
8/Lowered the credit card interest rates, minimum credit card payment, and late payment penalty.		

Source: IMF Country Report No. 21/46, International Monetary Fund [124].

Therefore, to investigate the causal effect of corporate ERM on its OE during the COVID-19 pandemic, the study defined the time windows to ensure that the changes in corporate solvency and liquidity ratios are indeed led by the COVID-19 pandemic. Hence, the study divided the sample period into three subperiods. The period from January 2020 to March 2020 was defined as an interim period; the period from October 2018 to December 2019 was defined as the pre-treatment period, and the period between April 2020 to March 2021 was defined as the post-treatment period. Two dummy variables were created, such as (i) Period is a dummy variable that takes the value of one when the period is between January 2020 to March 2021 (treatment period) and 0 if otherwise; (ii) PeriodQ12020 is a dummy variable which takes the value of one if the period is between January 2020 and March 2020 and 0 for otherwise.

In addition, to implement the DID analysis, each risk management variable (solvency and liquid) was divided into two groups (treatment group and control group). To do that, the study compared the average ratio of the risk management variable before (October 2018 to December 2019) to their average ratio during the crisis (January 2020 to March 2021). Thus, all the industrial sectors with an average ratio of solvency or liquidity during the COVID-19 pandemic (January 2020 to March 2021) higher than their average ratio before (October 2018 to December 2019) were set as the treatment group, and the otherwise as to the control group. Treatment is a new dummy variable that was created and takes the value of 1 if the average benchmark ratio of an industrial sector belongs to the treatment group and 0 if otherwise.

### 3.2 Operationalization of the variables

#### 3.2.1 Dependent variable: operational efficiency

Operational efficiency, yet a dimension of the usefulness of the ratio analysis, pertinent from the management's perception, is that it highlights the level of competence & effectiveness in the management and asset utilization. It indicates the efficiency of using business operation and solvency input elements. This concept differs among researchers and can be defined as how well a corporation employs its resources to generate profit [108]. Researchers [109] added that it reflects the operation results that a corporate possibly got when it used its input for business operations. The study of [110] argued that OE is a critical system that can keep a corporate in business or close it down. Relying on these definitions and due to the context of the COVID-19 pandemic, the study conceptualized the outcome variable as operational efficiency. In terms of measurement, operational efficiency has been treated as corporate performance and estimated by corporate financial ratios such as solvency, capital structure, profitability, and turnover [111]. Researchers [112] also treated OE as corporate performance measured by changes in stock market value, cash flow performance measured by solvency, and profitability measured by Return on investment (ROI). In the context of this study, the outcome variable operational efficiency was measured by corporate profitability ratios, return on asset (ROA) given in Eq (1)., and return on equity (ROE) given in Eq (2)., and the ratio of net income (annualized) over the total assets (OE) given in Eq (3). Following the equation (Eq) formulas of the study outcome variables.

(1) $${ROA}_{i,j,t}=\frac{{net\;income\;\left(annualized\right)}_{i,j,t}}{{average\;assets}_{i,j,t}}$$

(2) $${ROE}_{i,j,t}=\frac{{net\;income\;\left(annualized\right)}_{i,j,t}}{{average\;equity}_{i,j,t}}$$

(3) $${OE}_{i,j,t}=\frac{{net\;income\;\left(annualized\right)}_{i,j,t}}{{total\;assets}_{i,j,t}}$$

In variable measurement (1), (2), (3), i denotes individual corporations at time t in group j.

#### 3.2.2 Independent variable: effective risk management

Effective Risk Management (ERM) in this study is defined as a sophisticated management practice through debt restructuring and/or refinancing, but also through the accumulation of capital benefit from the government's temporary economic recovery policy on debt services and amortization relief that leads the corporation, the capability to hold a sufficient amount of cash (short-term and/or long-term) to fund its operation. A higher debt to equity (DE), given in Eq (4)., or debt to the asset (DA), given in Eq (5)., means the corporation has enough long-term cash to fund its operation and a higher current ratio (CR), given in Eq (6)., or quick ratio (QR), given in Eq (7)., means the corporation has enough short-term cash to fund its operation. However, lower DE/DA (Eqs (4), (5))) means the corporation has less long-term cash to fund its operation, and lower CR/QR (Eqs (6), (7))) means the corporation has less short-term cash to fund its operation. Following the equation formulas of the risk management variables.

(4) $${DE}_{i,s,t}=\frac{{Total\;Liabilities}_{i,s,t}}{{Total\;Equity}_{i,s,t}}$$

(5) $${DA}_{i,s,t}=\frac{{Total\;Liabilities}_{i,s,t}}{{Total\;Assets}_{i,s,t}}$$

(6) $${CR}_{i,s,t}=\frac{{Current\;Assets}_{i,s,t}}{{Current\;Liabilities}_{i,s,t}}$$

(7) $${QR}_{i,s,t}=\frac{{\left(Current\;Assets-Inventory\right)}_{i,s,t}}{{Current\;Liabilities}_{i,s,t}}$$

In variable measurement (4), (5), (6), (7), i denotes an individual corporation, s denotes the industrial sector that belongs to the corporation, and t, denotes the time or period. Table 4 presents a summary description of the study's independent variables.

**Table 4:** Summary of the study's independent variables.

Risk Management Variable	Risk Ratio/Variable	Variable scope	Variable Operationality
Solvency	DE	DE < AvDE	Less-leveraged
		DE ≥ AvDE	Leveraged
	DA	DA < AvDA	Less-leveraged
		DA ≥ AvDA	Leveraged
Liquidity	CR	CR < AvCR	Less-liquid
		CR ≥ AvCR	Liquid
	QR	QR < AvQR	Less-liquid
		QR ≥ AvQR	Liquid

Note: AvDE; AvDA; AvCR; and AvQR; denote the average debt-to-equity ratio; average debt-to-asset ratio; average current ratio; and average quick ratio, respectively.

### 3.3 Research model estimation

In order to investigate the causal effect of corporate effective risk management on its OE during the COVID-19 pandemic, this study applies the DID technique to compare changes over time in the treatment and control groups. Even under these circumstances, plausible assumptions often exist under which the study control for time-invariant differences in the treatment and control groups and estimates the causal effects of the intervention. Following this logic, the study designs the DID model as follows:

(8) $$Y_{i,j,t}=\partial_{j}+\delta_{t}+\beta_{1}Treatment+\beta_{2}Period+\beta_{3}\;Treatment\;*\;Period+U_{i,j,t}+\varepsilon_{i,j,t}$$

Where $\partial_{j}$ captures group-level time-invariant “fixed effects,” $\delta_{t}$ captures period time-invariant fixed effects. Treatment is a dummy variable that takes the value of 1 if the average ratio of solvency (DE or DA) or liquidity (CR or QR) during the post-treatment (period of the COVID-19 pandemic) is greater than the average ratio before and the value of 0 if otherwise (control group). Period is a dummy variable that takes the value of 1 marking the time of post-treatment, and 0, if otherwise. The interaction term between Treatment and Period (Treatment*Period) is the study's main variable of interest and its coefficient $\beta_{3}$ that measures the difference in solvency or liquidity between the treatment group (greater average ratio during the COVID-19 pandemic than before the COVID-19 pandemic) and the control group (those with a lower average ratio during the COVID-19 pandemic than before the COVID-19 pandemic). In contrast, $\beta_{2}$ measures the difference between the periods before and during the COVID-19 pandemic for the control group, and $\beta_{1}$ measures the difference between the treatment and control groups during the pre-COVID- 19 periods (before the COVID-19 pandemic). Hence, the DID coefficient $\beta_{3}$ removes biases in the post-treatment period comparison between the treatment and the control group that might be due to permanent differences between the control and the treatment groups, as well as biases resulting from comparisons over time in the treatment group that could be the result of trends. $U_{i,j,t}$ captures individual-level factors that vary across groups and over time, and $\varepsilon_{i,j,t}$ captures random error. The study also controls the macroeconomic variable that might affect corporate operational efficiency, among them the real GDP growth and inflation growth.

## 4 Research findings

### 4.1 Descriptive analysis findings

To identify the change in the corporate solvency and liquidity ratios, the study sample period was divided into two subperiods: the period before (from October 2018 to December 2019) and the period during COVID-19 (from January 2020 to March 2021). Descriptive statistics were applied to compare the mean of the risk management variables before and during the COVID-19 pandemic of each industrial sector for the eight industrial sectors that composed the Indonesian listed corporations. The findings show in Table 5 that the average benchmark solvency ratios of the industrial sectors, such as agriculture; basic industry; consumer goods; property; infrastructure, utility, and transportation, during COVID-19 are greater than those before or pre-COVID-19, making these industrial sectors leveraged.^2^ However, the average benchmark solvency ratio of industrial sectors, such as mining, miscellaneous, and trade, before COVID-19 is greater than during COVID-19, making these industrial sectors less-leveraged.^3^ Additionally, the average benchmark liquidity ratio of the industrial sectors, such as agriculture; mining; miscellaneous; and consumer goods, during the COVID-19 pandemic are greater than before the COVID-19 pandemic, making these industrial sectors liquid.^4^ But, the average ratio of the benchmark liquidity of the industrial sectors, such as basic industry; property; trade and infrastructure, utility, and transportation, before COVID-19 are greater than during COVID-19, making these industrial sectors less-liquid.^5^

**Table 5:** Summary Statistics of the risk management variables before and post-treatment.

	Pre-treatment, between October 2018 to December 2019 (before COVID-19 pandemic)															
	Agriculture Sector				Mining Sector				Basic Industry Sector				Miscellaneous industry Sector			
	Mean	SD	Min	Max	Mean	SD	Min	Max	Mean	SD	Min	Max	Mean	SD	Min	Max
DE	1.21	0.04	1.18	1.27	1.38	0.08	1.25	1.46	1.15	0.04	1.10	1.21	1.22	0.04	1.16	1.27
DA	0.55	0.01	0.54	0.56	0.58	0.01	0.56	0.59	0.53	0.01	0.52	0.55	0.55	0.01	0.54	0.56
CR	0.92	0.08	0.86	1.04	1.25	0.02	1.23	1.28	1.57	0.11	1.38	1.65	1.21	0.05	1.13	1.25
QR	0.60	0.07	0.54	0.69	1.05	0.02	1.03	1.08	1.07	0.06	0.98	1.13	0.90	0.04	0.83	0.94
	Consumer goods industry sector				Property Sector				Infrastructure, Utility, and transportation				Trade Sector			
	Mean	SD	Min	Max	Mean	SD	Min	Max	Mean	SD	Min	Max	Mean	SD	Min	Max
DE	0.71	0.03	0.65	0.73	1.15	0.06	1.08	1.23	1.69	0.07	1.58	1.77	0.93	0.07	0.86	1.03
DA	0.41	0.01	0.39	0.42	0.53	0.01	0.52	0.55	0.63	0.01	0.61	0.64	0.48	0.02	0.46	0.51
CR	1.83	0.08	1.70	1.90	1.67	0.02	1.64	1.70	0.70	0.04	0.64	0.74	1.45	0.04	1.39	1.51
QR	1.01	0.05	0.92	1.06	1.11	0.06	1.03	1.18	0.66	0.04	0.61	0.71	1.03	0.04	0.98	1.09
Post-treatment, between January 2020 to March 2021 (During COVID-19 pandemic)																
	Agriculture Sector				Mining Sector				Basic Industry Sector				Miscellaneous industry Sector			
	Mean	SD	Min	Max	Mean	SD	Min	Max	Mean	SD	Min	Max	Mean	SD	Min	Max
DE	1.35	0.07	1.28	1.45	1.27	0.12	1.13	1.45	1.17	0.02	1.15	1.20	1.09	0.06	1.03	1.19
DA	0.57	0.01	0.56	0.59	0.56	0.02	0.53	0.59	0.54	0.01	0.53	0.55	0.52	0.01	0.51	0.54
CR	0.95	0.09	0.84	1.05	1.37	0.02	1.35	1.41	1.56	0.03	1.53	1.60	1.31	0.03	1.27	1.35
QR	0.65	0.07	0.55	0.72	1.19	0.04	1.16	1.26	1.12	0.02	1.09	1.14	1.01	0.05	0.93	1.06
	Consumer goods industry sector				Property Sector				Infrastructure, Utility, and transportation				Trade Sector			
	Mean	SD	Min	Max	Mean	SD	Min	Max	Mean	SD	Min	Max	Mean	SD	Min	Max
DE	0.78	0.08	0.69	0.87	1.25	0.07	1.18	1.35	2.24	0.17	2.03	2.48	0.90	0.05	0.83	0.97
DA	.44	.02	.41	.46	.554	.02	.54	.57	.69	.02	.67	.71	.47	.02	.45	.49
CR	1.97	0.07	1.93	2.09	1.48	0.08	1.39	1.59	0.61	0.04	0.58	0.67	1.38	0.03	1.35	1.40
QR	1.13	0.06	1.09	1.23	0.85	0.05	0.79	0.93	0.59	0.04	0.56	0.64	1.00	0.03	0.97	1.04

This table compares the summary statistics of the risk management variables (benchmark solvency and liquidity ratios) during the COVID-19 pandemic (January 2020 to March 2021) to before the COVID-19 pandemic (October 2018 to December 2019. The number of observations in each industrial sector is five, both Pre-treatment and Post-treatment. All the variables are expressed in percentages (%).

The average ratio of the benchmark solvability and liquidity ratios of the trade industrial sector are both greater before COVID-19 than during the COVID-19 period, making this industrial sector the most vulnerable^6^ during the COVID-19 pandemic. However, the average ratio of benchmark solvability and liquidity of agriculture and consumer goods industry sectors during COVID-19 are greater than before COVID-19, making these industries the most resilient^7^ to the COVID-19 pandemic. These findings come in support of the previous empirical findings of researchers [113,114], who argued that the impact of the COVID-19 pandemic on corporate financial indicators has been unequal across different sectors of industry; and also, in support of the previous work by researchers [[115], [116], [117], [118]] who concluded their studies that the NFCs resilience to the COVID-19 depends on their pre-COVID-19 characteristics such as cash (solvability and liquidity), and profitability.

### 4.2 Graphical analysis findings

In this subsection, the study further applied a graphical analysis to help convey the causal effect of ERM practices from credit supply shocks due to the financial crisis led by the COVID-19 pandemic on NFCs. Fig. 4a, b and 4c illustrate the trend of the study outcome variables (ROA, ROE, and OE).

In Fig. 4a, b, and 4c, the agriculture and consumer goods industrial sectors show a progressive improvement trend of ROA, ROE, and OE after the shock. These findings are consistent with the results in Table 5, which indicated that NFCs with higher solvency and liquidity ratio during COVID-19 than before COVID-19 are the most resilient industrial sector. This finding lends initial support to the applied risk management theory, which emphasizes that ERM helps NFCs match the maturity of the assets and liabilities and offers protection from credit supply shocks, such as having to refinance long-term debt in bad situations. However, it is too early to conclude at this stage.

Additionally, the Mining and Miscellaneous industrial sectors show a steady improvement trend of ROA, ROE, and OE after the shock but with a lower magnitude than the resilient industrial sector. This indicated that NFCs with a higher liquidity ratio during COVID-19 than before COVID-19 (liquid corporation) have a positive association with operational efficiency. This finding lends initial support to the study hypothesis one. However, it is too early to conclude at this stage.

Moreover, the Basic industrial; property; infrastructure, utilities, and transportation; improvements have been damped or reversed the ROA, ROE, and OE trend after the shock. These findings indicated that NFCs with a higher solvency ratio during COVID-19 than before COVID-19 (leveraged corporations) have a negative association with operational efficiency. This finding lends initial support to the study hypothesis two. However, it is too early to conclude at this stage.

### 4.3 Baseline analysis findings

To analyze how the solvability and liquidity of Indonesian listed corporations from the different industrial sectors change affect their OE following the COVID-19 pandemic, the study compares their average ratio before and during the COVID-19 pandemic and implements DID estimates on them. The results are presented in Table 6.

**Table 6:** Effect of the COVID-19 Pandemic, profitability, and operational efficiency.

	(1)	(2)	(3)	(4)	(5)	(6)
	ROA	ROA	ROE	ROE	OE	OE
Treatment_SV	0.0858 (1.086)		1.236 (1.932)		0.000797 (0.0106)	
Treatment_LQ		−2.274* (1.087)		−3.605 (1.977)		−0.0219* (0.0106)
Period	0.339 (1.683)	−3.983* (1.772)	0.592 (2.994)	−7.439* (3.225)	0.00381 (0.0165)	−0.0383* (0.0174)
Treatment_SV * Period	−4.086** (1.512)		−8.812** (2.690)		−0.0401** (0.0148)	
Treatment_LQ * Period		4.580** (1.537)		7.558** (2.796)		0.0443** (0.0151)
GDP	−0.00943 (0.207)	−0.00943 (0.214)	−0.0365 (0.369)	−0.0365 (0.390)	−0.000148 (0.00203)	−0.000148 (0.00210)
Inflation	0.149 (0.295)	0.149 (0.305)	0.354 (0.525)	0.354 (0.554)	0.00167 (0.00288)	0.00167 (0.00299)
_cons	2.854 (2.613)	4.023 (2.721)	5.009 (4.648)	7.276 (4.952)	0.0263 (0.0255)	0.0376 (0.0267)
*N*	80	80	80	80	80	80
*R*^2^	0.239	0.188	0.287	0.204	0.241	0.186

This table presents DID estimates of the effect of the COVID-19 pandemic on the corporate profitability and operational efficiency of the leveraged and liquidity sectors relative to the less-leveraged and less-liquidity sectors. The independent variables in models (1) to (4) are profitability ratios, respectively net income (annualized) over the average assets (ROA) and net income (annualized) over the average equity ratio (ROE), and the independent variables in models (5) to (6) is operational efficiency ratio (OE) given by net income (annualized) over the total assets. Treatment_Solvability is a dummy variable marking all corporates belonging to the sector that the average benchmark solvability ratio during the COVID-19 pandemic (January 2020 to March 2021) is higher than before the COVID-19 pandemic. Treatment _ Liquidity is a dummy variable marking all corporates belonging to the sector that the average of the liquidities ratios during the COVID-19 pandemic (January 2020 to March 2021) is higher than before the COVID-19 pandemic (October 2018 to December 2019). Period is a dummy variable that takes the value of 1 if the period is between January 2020 to March 2021 (treatment period) and 0 if otherwise (control period). The macro-level variable (GDP and inflation) controls. The lower part denoted the number of observations and adjusted R2. Standard errors are reported in parentheses, *p < 0.05, **p < 0.01, ***p < 0.001. All the indicators are expressed in percentages (%).

The DID estimate, which is the interaction term between Treatement_LQ and Period (Treatement_LQ*Period), the study's main variable of interest presents a positive and significant coefficient for the outcome variable, ROE, 7.558 significant at 1%. This finding is consistent across the outcome variable, ROA, with DID coefficient of 4.580 significant at 1%. The same scenario is observed on the outcome variable OE (DID coefficient, 0.0443 significant at 1%) but with a less increase in magnitude compared to ROE or ROA. The extent of these coefficients shows that the operational efficiency of liquid corporations significantly rose by 7.558, 4.580, and 0.0443, respectively, for the outcome variables ROE, ROA, and OE, during the COVID-19 pandemic.

The findings are also consistent with the results in subsection 4.2, which indicated that NFCs with a higher liquidity ratio during COVID-19 than before COVID-19 (liquid corporation) have a positive association with operational efficiency. Therefore, hypothesis 1 is supported. These findings are consistent with previous empirical studies, which indicated that short-term debt raises OE as they lessen investor risk, an aspect that alternates more restrictive credit contracts [56,57,[67], [68], [69], [70], [71]].

The interaction term between Treatement_SV and Period (Treatement_SV*Period), the study's main variable of interest, presents a negative and significant coefficient for the outcome variable, ROE, −8.812 significant at 1%. This finding is consistent across the outcome variable, ROA, with DID coefficient of −4.086, significant at 1%. The same scenario is observed on the outcome variable OE (DID coefficient, −0.0401 significant at 1%) but with a slight decrease in magnitude compared to ROE or ROA. The range of these coefficients shows that the operational efficiency of leveraged corporations significantly decreased by −8.812, −4.086, and 0.0401, respectively, for the outcome variables ROE, ROA, and OE, during the COVID-19 pandemic.

These findings are consistent with the results in subsection 4.2, which indicated that NFCs with a higher solvency ratio during COVID-19 than before COVID-19 have a negative association with operational efficiency. Hence, since the treatment group observations were collected during a short-term investment period (from January 2020 to March 2021), hypothesis 2 is supported.

This finding can be enlightened by the fact that better NFCs that applied ERM practices prefer to use short-term debt to fund their working capital and short-term investment project to take advantage of future disclosure of positive information, as suggested by Ref. [119] for the NFCs at the upper end of the quality spectrum. For example, NFCs encounter financial distress due to a lack of liquidity (short-term insolvency) and bankruptcy due to the lack of long-term debt. While there is risk associated with continually refinancing liquidity with short-term debt due to the credit supply shocks and since short-term debt requires the NFCs to refinance frequently, it increases the corporate short-term debt refinancing failure costs according to Ref. [120] because often lenders do not wish to refinance short-term debt when it matures [84]. Thus, the threat of financial distress and the requirements of short-term lenders motivate managers to achieve positive and significant operational efficiency with liquidity. These results are similar to the findings of [75,82], who suggest that the differences in corporate debt maturity had real important effects on short-term and long-term investment projects.

### 4.4 Common trend assumption

According to Ref. [91], contemporary researchers' applications of the DID design should devote much attention to statistical analysis (sensitivity analysis and robustness analysis) and graphical analysis to checks designed to probe the main assumptions that support the internal validity of the research design. Although the specific details involved vary with the context and data limitations of individual studies, the following three subsections provide the findings of both statistical and graphical check that was used in the current study to shed light on the validity of the common trend's assumption and threats to the strict exogeneity condition.

#### 4.4.1 Cross-sectional with leveraged and liquid corporations findings

While the baseline findings present the operational efficiency of the leveraged and liquid corporation during the COVID-19 pandemic, the change in these ratios might lead management to implement certain risk management practices. For example, the manager may adopt a different risk management strategy for different financial risk indicators. Thus, the study explores the cross-sectional analysis along with the leveraged and liquid corporations. To carry out this analysis, the study estimates the difference-in-difference-in-difference (DDD) equation in which the original DID interaction term interacts with the leveraged or liquid corporation's variable. The results are shown in Table 7.

**Table 7:** Effect of the COVID-19 Pandemic, profitability, and operational efficiency by corporation industrial sector.

	(1)	(2)	(3)	(4)	(5)	(6)
	ROA	ROA	ROE	ROE	OE	OE
Treatement_SV	0.0858 (1.080)		1.236 (1.919)		0.000797 (0.0106)	
Treatement_LQ		−2.274* (0.889)		−3.605* (1.608)		−0.0219* (0.00877)
Period	−0.133 (0.992)	−4.455*** (0.889)	−0.433 (1.763)	−8.463*** (1.608)	−0.00119 (0.00970)	−0.0433*** (0.00877)
Treatement_SV*Period	−4.688* (1.998)		−10.55** (3.552)		−0.0460* (0.0195)	
Treatement_LQ* Period		−2.575 (1.733)		−5.665 (3.134)		−0.0250 (0.0171)
Treatement_SV*Period*Leveraged	0.142 (0.310)		0.410 (0.551)		0.00139 (0.00303)	
Treatement_LQ*Period*Liquid		2.385*** (0.397)		4.408*** (0.719)		0.0231*** (0.00392)
_cons	4.216*** (0.661)	5.385*** (0.628)	8.175*** (1.175)	10.44*** (1.137)	0.0414*** (0.00647)	0.0526*** (0.00620)
*N*	80	80	80	80	80	80
*R*^2^	0.238	0.449	0.288	0.467	0.239	0.441

This table compares DID estimates of the effect of the COVID-19 pandemic on the corporate operational efficiency by the industry sector with leveraged and liquidity. We checked the effect of COVID-19 from January 2020 up to March 2021. The independent variables in models (1) to (4) are profitability ratios, respectively net income (annualized) over the average assets (ROA) and net income (annualized) over the average equity ratio (ROE), and the independent variables in models (5) to (6) is operational efficiency ratio (OE) given by net income (annualized) over the total assets. Treatment_Solvability is a dummy variable marking all corporates belonging to the sector that the average leveraged ratio during the COVID-19 pandemic (January 2020 to March 2021) is higher than before the COVID-19 pandemic. Treatment_ Liquidity is a dummy variable marking all corporates belonging to the sector that the average of the liquidities ratios during the COVID-19 pandemic (January 2020 to March 2021) is higher than before the COVID-19 pandemic (October 2018 to December 2019). Period is a dummy variable that takes the value of 1 if the period is between January 2020 to March 2021 (treatment period) and 0 if otherwise (control period). The lower part denoted the number of observations and adjusted R2. Standard errors are reported in parentheses, *p < 0.05, **p < 0.01, ***p < 0.001. All the indicators are expressed in percentages (%).

The study's main interest term in this section is the DDD estimate. I.e., the interaction term between the original DID interaction term and the leveraged or liquid corporations variable. The findings show positive DDD estimates for all outcome variables (ROE, ROA, and OE). This implies that corporate managers have raised their liquidity and solvability capability following the persistence of the COVID-19 pandemic. However, the DDD estimates of the liquid corporations (Treatment_SV*Period*Liquid), 4.408, 2.385, and 0.023, respectively, for the outcome variable ROE, ROA, and OE are all significant at 0.1%, while the DDD estimates of the leveraged corporations (Treatment_SV*Period*Leveraged), 0.410, 0.142, and 0.00139 respectively for the outcome variable ROE, ROA, and OE are all non-significant. This means that when the COVID-19 pandemic persists, the manager tends to raise the corporate liquidity stock much higher than its solvability by debt refinancing and/or debt restructuring, but also the accumulated capital benefits from government temporary recovery policy on debt services and amortization relief. This is naturally the risk management practice leading corporations to easier meet their financial obligations in the bad situation and continuity in the industry, as failure to do that could lead the corporate to face bankruptcy.

The findings are consistent with the results in subsection 4.2, which indicated that NFCs with higher solvency and liquidity ratio during COVID-19 than before COVID-19 showed a progressive improvement trend of ROA, ROE, and OE after the shock. Thus, these findings support the risk management theory applied, which suggests that ERM is an ongoing process that requires the corporation to apply adequate security measures to diminish the threats. Because the results show that adequate securities measures with debt refinancing and/or debt restructuring help NFCs secure enough liquidity by matching the maturity of their assets and liabilities, it also offers protection to NFCs from credit supply shocks due to the financial crisis triggered by the COVID-19.

These findings also refer to the fact that NFCs have implemented a sophisticated risk evaluation process of probing what might cause damage during the period of activity, which helped them to take sufficient risk management measures to minimize the risk as much as possible to prevent the corporation from bankruptcy and continuing in the industry while operating efficiently [39]. One possible reason for the indebted corporation is that financial pressure may force them and their managers to be more efficient. These findings are further supported by the conclusion of [64], who argued that corporates renegotiate their credit maturity with their lender to execute credit repayment on the business since the increase in liquidity during the crisis could result from debt refinancing and/or debt restructuring, but also through the cumulated capital benefit from debt services and amortization relief. Moreover, it aligned with the conclusion of [66], who suggested that debt refinancing and/or restructuring is associated with corporate OE in the use of the assets.

#### 4.4.2 Robust analysis findings

Given that there are around three months or one quarter (Q1, 2020) that separate the discovery of COVID-19 in Wuhan, China, from the Indonesian first case, the study implements a multiple DID analysis to offer an additional sensitivity test. Thus, we integrated all three periods of time into the analysis, including the pre-treatment period from October 2018 to December 2019, the interim period of January 2020 to March 2020, and the post-treatment period from April 2020 to March 2021. The results are shown in Table 8.

**Table 8:** Effect of the COVID-19 pandemic, profitability, and operational efficiency, multiple-period DID analysis.

	(1)	(2)	(3)	(4)	(5)	(6)
	ROA	ROA	ROE	ROE	OE	OE
Treatement_SV	0.0858 (1.086)		1.236 (1.933)		0.000797 (0.0106)	
Treatement_LQ		−2.274* (1.086)		−3.605 (1.973)		−0.0219* (0.0106)
Period	−0.351 (1.064)	−4.683*** (1.152)	−0.865 (1.895)	−9.074*** (2.092)	−0.00352 (0.0104)	−0.0456*** (0.0113)
Treatement_SV*Period	−3.883* (1.600)		−8.473** (2.849)		−0.0380* (0.0156)	
Treatement_LQ*Period		4.803** (1.629)		8.253** (2.959)		0.0464** (0.0160)
PeriodQ12020	1.091 (1.858)	1.143 (1.920)	2.162 (3.309)	3.053 (3.487)	0.0117 (0.0182)	0.0114 (0.0188)
Treatement_SV*PeriodQ12020	−1.012 (2.628)		−1.695 (4.679)		−0.0109 (0.0257)	
Treatement_LQ*PeriodQ12020		−1.116 (2.715)		−3.476 (4.932)		−0.0103 (0.0266)
_cons	4.216*** (0.665)	5.385*** (0.768)	8.175*** (1.184)	10.44*** (1.395)	0.0414*** (0.00650)	0.0526*** (0.00753)
*N*	80	80	80	80	80	80
*R*^2^	0.240	0.189	0.287	0.208	0.241	0.186

This table presents multiple periods of DID estimates of the effect of the COVID-19 pandemic on the corporate profitability and operational efficiency of the leveraged and liquidity sectors relative to the less-leveraged and less-liquidity sectors. The independent variables in models (1) to (4) are profitability ratios, respectively, net income (annualized) over the average assets (ROA) and net income (annualized) over the average equity ratio (ROE), and the independent variables in models (5) to (6) is operational efficiency ratio (OE) given by net income (annualized) over the total assets. Treatment_Solvability is a dummy variable marking all corporates belonging to the sector that the average solvability ratio during the COVID-19 pandemic (January 2020 to March 2021) is higher than before the COVID-19 pandemic. Treatment _ Liquidity is a dummy variable marking all corporates belonging to the sector that the average of the liquidities ratios during the COVID-19 pandemic (January 2020 to March 2021) is higher than before the COVID-19 pandemic (October 2018 to December 2019). Period is a dummy variable that takes the value of 1 if the period is between January 2020 to March 2021 (treatment period) and 0 if otherwise (control period); PeriodQ12020 is a dummy variable that takes the value of 1 if the period is between January 2020 and March 2020 and 0 if otherwise. The lower part denoted the number of observations and adjusted R2. Standard errors are reported in parentheses, *p < 0.05, **p < 0.01, ***p < 0.001. All the indicators are in percentage (%).

While the DID estimates resulting from the interaction term between treatment and Period are consistent with the original DID estimates in the baseline analysis, the DID estimates resulting from the interaction term between treatment and the interim period variable (PeriodQ12020) at all outcome variables are negative and non-significant. This means that corporates did not adjust their risk management strategy until the country announced its first case of COVID-19, followed by the WHO announcement of COVID-19 as a pandemic. In addition, since the post-treatment period (from April 2020 to March 2021) is one year counted, hence the short-term period, these findings are therefore robust and consistent with reality. Thus, this indicated that corporate risk management during COVID-19 is the source of structural change, which affects its existence and operational efficiency.

#### 4.4.3 Graphical validation of the common trend assumption

**Fig. 2:**
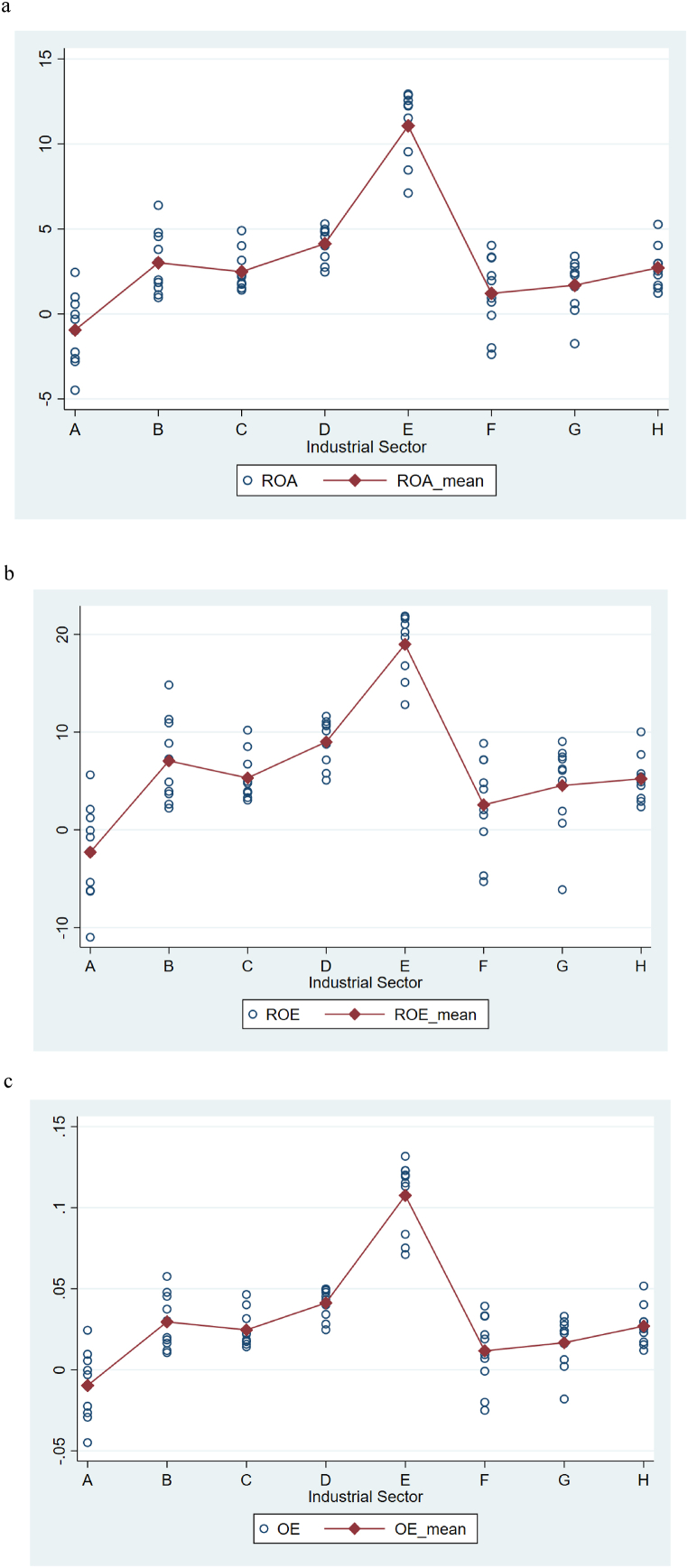
aPeriod time-invariant Fixed effects: Heterogeneity across Industrial Sector for the outcome variable ROA (unobserved variables that do not change over time). Period time-invariant Fixed effects: Heterogeneity across Industrial Sector for the outcome variable ROE (unobserved variables that do not change over time). Period time-invariant Fixed effects: Heterogeneity across Industrial Sector for the outcome variable OE (unobserved variables that do not change over time).

**Fig. 3:**
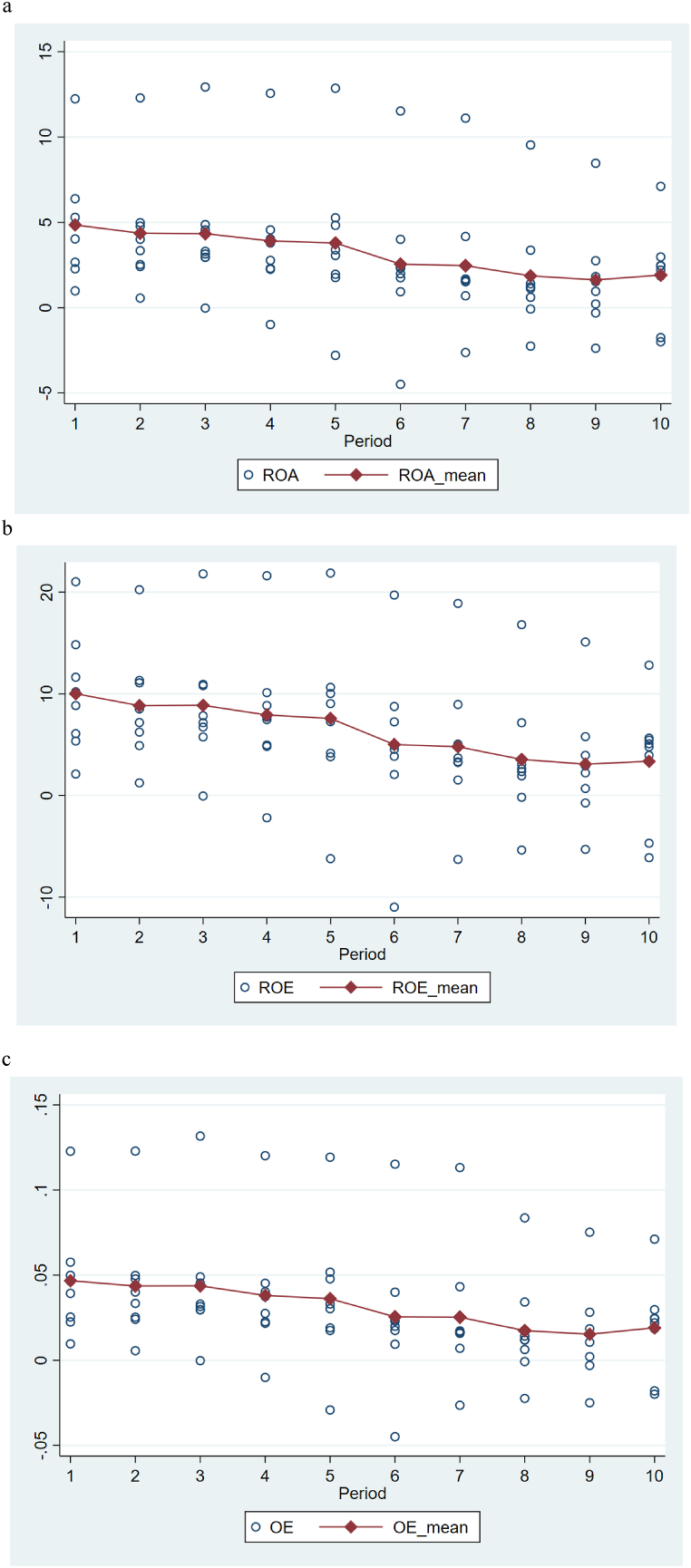
aGroup-level time-invariant Fixed effects: Heterogeneity across Period for the outcome variable ROA (unobserved variables that do not change over time). Group-level time-invariant Fixed effects: Heterogeneity across Period for the outcome variable ROE (unobserved variables that do not change over time). Group-level time-invariant Fixed effects: Heterogeneity across Period for the outcome variable OE (unobserved variables that do not change over time).

A graph of the time series should look like a set of parallel lines [91]. They also added that parallel lines do not have to be linear: Time-fixed effects allow for flexible time trends that move up or down from period to period, as they do. In the current paper, the study plots the mean outcomes by group and period, and as a result, the lines appear to be approximately parallel in Fig. 2, Fig. 3c illustrate respectively; the period time-invariant fixed effects (Heterogeneity across Industrial Sector) and period time-invariant fixed effects (Heterogeneity across Industrial Sector) for the outcome variable ROA, ROE, and OE. Therefore, we concluded that the common trend assumption is verified in the current study.

**Fig. 4:**
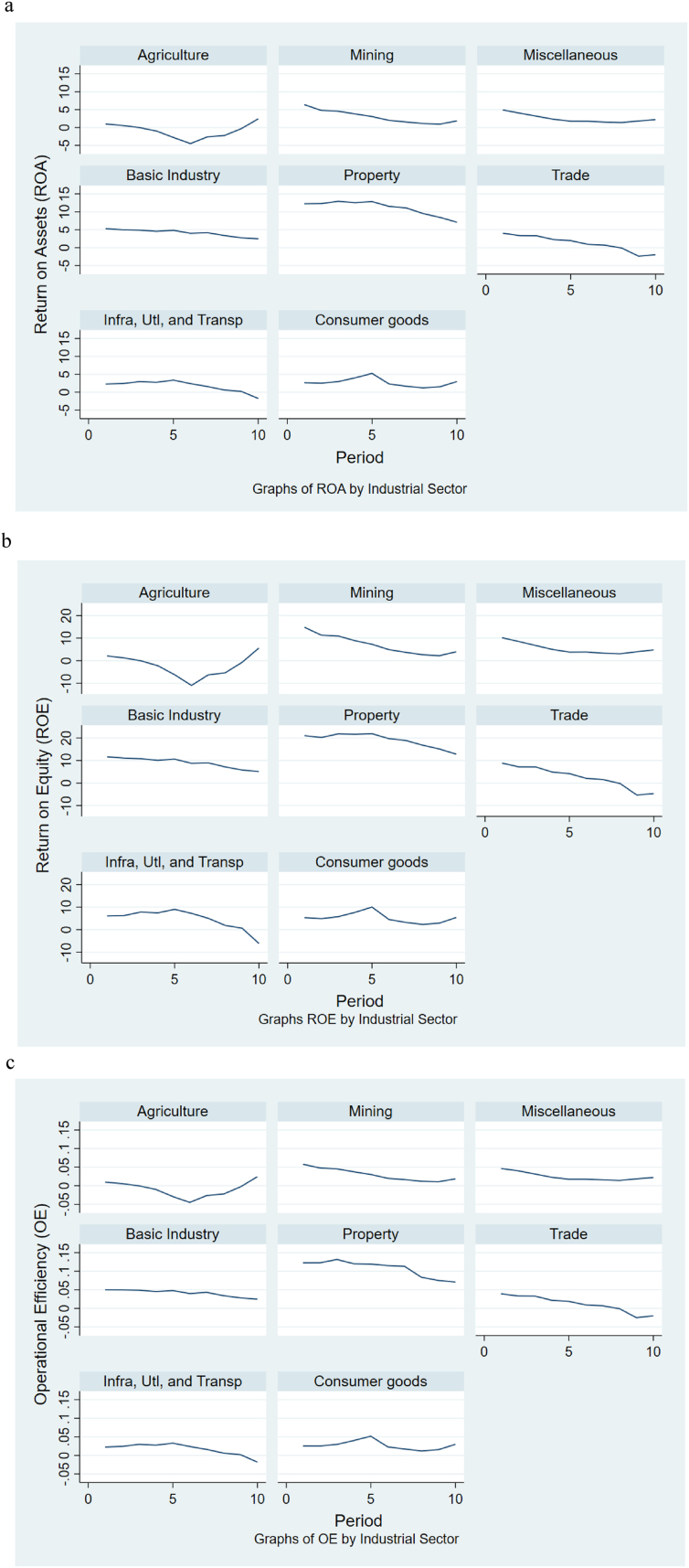
aFix effects of ROA by Industrial Sector. Fix effects of ROE by Industrial Sector. Fix effects of OE by Industrial Sector.

## 5 Conclusion

To investigate the causal effect of corporate ERM practices on corporate OE during COVID-19, this study used the COVID-19 pandemic to identify the treatment group as the difference in change in the NFCs risk management ratios over time. A thorough quantitative analysis including the descriptives, baseline, cross-sectional, robust check, and graphical analyses grounded on the DID technique. Especially a quasi-experiment was applied to compute the empirical analysis of the data of Indonesian listed corporations collected from the central bank of Indonesia (Bank of Indonesia), and the risk management theory was further applied to help discuss the result.

The findings suggest that the operational efficiency of the liquid corporations during the COVID-19 pandemic significantly increased compared to the pre-treatment period. Meanwhile, the operational efficiency of the leveraged corporations during the COVID-19 pandemic significantly decreased compared to the pre-treatment period. This indicates that corporations implementing ERM during the COVID-19 pandemic to raise their long-term debt financing capability significantly decreases their operational efficiency. However, those who applied it aiming to raise their liquidity capability generated a positive and significant effect on their operational efficiency. These findings align with those of [84], who argued that the maturity of the long-term debt corresponds to the maturity of cash flows from a company's investments and which must be refinanced on terms that depend on the credit rating at the time of its renewal, while short-term debt has a maturity before the cash flows arrive from a firm's investments. Moreover, they added that short-term debt (liquidity) is preferable for working capital; therefore, OE will be negatively associated with long-term debt financing.

Furthermore, the DDD analysis refined these findings by highlighting that corporate during the COVID-19 period tended to increase their liquidity stock higher than the solvability so that they could be able to meet their financial obligations and continue staying in the industry since failure to do so could force them to face financial distress and bankruptcy. But the robust check also revealed that the DID estimates during the post-treatment period, the short-term (from April 2020 to March 2021), are consistent with the DID estimates in the baseline analysis during the COVID-19 period (from January 2020 to March 2021). Hence, the study's empirical findings are consistent with the applied risk management theory, which emphasizes that ERM leads NFCs to match the maturity of their assets and liabilities and offers protection from credit supply shocks, for example, having to refinance long-term debt in bad situations.

These findings come in support of existing literature that links OE with corporate risk management policy with credit maturity and aligned with the conclusion of [64], who argued that corporations refinance their debts to execute credit repayment on the business for several reasons, among them, is reducing the interest rates on loans, changing the loan structure, consolidating debts, and freeing up cash. Indebted corporations with high solvency ratios could benefit from debt refinancing since they can secure more satisfactory contract terms and lower interest rates. These findings also match the conclusion of [66], who showed that these measures are associated with corporate OE using the assets. The study, therefore, deducted that while debts amount and age may affect corporate credit score, ERM practices led to the indebted corporation the flexibility of debt refinancing or/and restructuring, which offer them the capability to prevent bankruptcy and adapt to the changes while operating efficiently” in the time of crisis.

Finally, the solid findings of this research suggest that corporate risk management during the COVID-19 pandemic is the source of structural change, which affects its existence and operational efficiency. The interim period served as an alert because corporate managers within the treatment group were aware of the enduring health crisis and its impact and could anticipate an ERM strategy. In such a situation, urgent action is needed to prevent the corporation from facing bankruptcy, but a sophisticated risk management strategy is important to lead the corporation toward a smooth transition while operating efficiently.

### 5.1 Study limitation

Non-financial corporations apply ERM practices that help them to match the maturity of the assets and liabilities, and thus they usually use long-term debt to make long-term investments, such as acquisitions of fixed assets or equipment. Long-term debt also offers protection from credit supply shocks and having to refinance in bad situations like the COVID-19 pandemic. Thus, while the empirical analysis provided is a useful first step, it should be emphasized that this paper does not address the ultimate question of whether, in a period of crisis like the COVID-19 pandemic, the analysis of long-term debt implications on the corporate OE should be the same as short-term debt and, if not, what should be the best way to do so? The question is complex because the consequences of distortions generated by this miss interpretation must be compared with capital market imperfections due to information problems that would exist without government controls.

While Corporate ERM practices aim to raise the long-term debt to improve productivity growth and capital accumulation, government policy in collaboration with their central and commercial bank to promote debt refinancing and/or restructuring often serves a purpose, such as correcting regional disproportions or endorsing greater equity in income distribution, which are not discussed in the present study. Finally, though the focus of this study is narrow, the available data does not provide the conditions and amount of long-term debt used for debt refinancing and/or restructuring and how government-supported temporary regulatory relief policy affects the corporate OE. In particular, there should be optimally detailed data on firm-level information on the amount of debt (long-term and short-term) refinanced or restructured, as well as information on the terms and conditions of each loan and the rates. Nonetheless, the empirical analysis of the determinants and consequences of the debt maturity structure presented in this study is a useful first step that highlights some of the interesting issues and problems in debt refinancing and/or restructuring that are relevant ERM practices that offers protection to NFCs from credit supply shocks during the COVID-19 pandemic.

### 5.2 Future study directions

The future research directions of the present paper are to study the debt maturity structure for Indonesian corporations before, during, and after the COVID-19 pandemic. The study will also discuss how the NFCs debt maturity structure has been affected by government temporary regulatory relief policies implemented to help restart the economic systems after the deep crisis brought on by the lockdown. Using micro-level panel data (firm-level data), the study will analyze how access to debt is associated with various corporate characteristics. Finally, the study will provide additional quantitative analysis on the impact of access to long-term debt on corporate productivity, capital accumulation, and growth.

## Author contribution statement

Jun Huang: Conceived and designed the analysis, Analyzed and interpreted the data.

Bienmali Kombate: Conceived and designed the analysis, Analyzed and interpreted the data, and Wrote the paper.

Yun Li: Analyzed and interpreted the data and Wrote the paper.

Konan Richard Kouadio: Contributed analysis tools or data, Analyzed and interpreted the data.

Peijun Xie: Contributed analysis tools or data.

## Data availability statement

Data will be made available on request.

## Declaration of interest's statement

The authors declare that they have no known competing financial interests or personal relationships that could have appeared to influence the work reported in this paper.

## Additional information

Supplementary content related to this article has been published online at [URL].

## Footnotes

1 Bank of Indonesia https://www.bi.go.id/en/statistik/ekonomi-keuangan/sski/default.aspx.

2 Leveraged means corporation has enough long-term cash to fund its operation.

3 Less-leveraged means corporation has less long-term cash to fund its operation.

4 Liquid means corporation has enough short-term cash to fund its operation.

5 Less-liquid mean corporation has less short-term cash to fund its operation.

6 Vulnerable means corporation have less cash (short-term and long-term) to fund its operation.

7 Resilient means corporation has enough cash (short-term and long-term) to fund its operation.
